# Body size affects the strength of social interactions and spatial organization of a schooling fish (*Pseudomugil signifer*)

**DOI:** 10.1098/rsos.161056

**Published:** 2017-04-26

**Authors:** Maksym Romenskyy, James E. Herbert-Read, Ashley J. W. Ward, David J. T. Sumpter

**Affiliations:** 1 Department of Mathematics, Uppsala University, PO Box 480, Uppsala 75106, Sweden; 2 Department of Zoology, Stockholm University, Stockholm 10691, Sweden; 3 School of Biological Sciences, University of Sydney, Sydney, New South Wales, Australia

**Keywords:** collective motion, interactions, statistical mechanics, fish school

## Abstract

While a rich variety of self-propelled particle models propose to explain the collective motion of fish and other animals, rigorous statistical comparison between models and data remains a challenge. Plausible models should be flexible enough to capture changes in the collective behaviour of animal groups at their different developmental stages and group sizes. Here, we analyse the statistical properties of schooling fish (*Pseudomugil signifer*) through a combination of experiments and simulations. We make novel use of a Boltzmann inversion method, usually applied in molecular dynamics, to identify the effective potential of the mean force of fish interactions. Specifically, we show that larger fish have a larger repulsion zone, but stronger attraction, resulting in greater alignment in their collective motion. We model the collective dynamics of schools using a self-propelled particle model, modified to include varying particle speed and a local repulsion rule. We demonstrate that the statistical properties of the fish schools are reproduced by our model, thereby capturing a number of features of the behaviour and development of schooling fish.

## 1. Introduction

In sufficiently large collective systems, the behaviour of an individual can be dominated by the generic statistical effects of many individuals interacting, rather than its own behaviour [1]. Much of the progress in understanding collective motion of animal groups has involved applying ideas borrowed from the statistical physics of materials like magnets or fluids [2–6]. For example, changes in group densities produce phase transitions at critical group sizes [7,8]. More complex collective states, such as swarms, mills and polarized groups, depend on the density of a group and the noise within the system [9]. Recent studies of starlings and midges have looked at spatial velocity fluctuations [10,11], long-range correlations [12], and diffusive [13] and entropic characteristics of flocks [14]. Other experiments with artificial particles have looked for similarities and differences between self-organized living matter and thermal equilibrium systems [15]. These latter approaches gather statistical information about self-organizing structures in order to parametrize models (see, for example, the maximum entropy approach [11,16]). However, none of these have explicitly solved the inverse problem of using the macro-level properties of animal groups to find out how the individuals within them interact.

This inference problem is essentially a statistical physics problem. The last few decades have seen a major increase in research at the interface of molecular dynamics and biophysics. In soft matter systems, estimating the potential energy of an interaction and the corresponding potentials is of particular importance, as the strength of intermolecular interactions determines the state of matter and many of its properties [17]. At the same time, molecular interaction potentials are difficult to measure experimentally and hard to compute from first principles. An alternative approach, therefore, is to estimate them from experimentally determined structures of molecules. The interactions in these structures are usually strongly coupled and assemblies are typically driven by weak forces (e.g. hydrophobicity or entropy) [18]. Therefore, estimation of these potentials requires application of sophisticated coarse-grained techniques such as reverse Monte Carlo [19], inverse Monte Carlo [20] or iterative Boltzmann inversion [21]. These methods adjust the force field iteratively, until the distribution functions of the reference system are reproduced as accurately as possible. In other cases, when the potentials are uncoupled or weakly coupled, a more straightforward direct Boltzmann inversion approach can be applied, which approximates the potential by the negative logarithm of the radial distribution function [22]. In collectively moving animal groups, the interactions between members are usually assumed to be of hierarchical structure, with repulsion having highest priority at small distances [23]. Thus, one can expect that the latter method can be also applied to animal self-organized systems, such as fish schools, to infer the interactions within these groups from experimental data.

Here, we investigate the schooling behaviour of fish using Boltzmann inversion and related methods. Unlike molecules and physical particles, fish change their behaviour as they go through various developmental stages [24]. For example, onset of schooling is only possible when the central nervous system of fish is sufficiently developed to support a high level of coordination of visual and mechanosensory information [25]. The developmental differences are usually also reflected in changes of the key characteristics of motion, including speed. Therefore, fish of different sizes cannot be considered simply as particles of different physical size, since their behaviour changes with their size. We thus expect the statistical properties of the group, and of individuals, to change both with the density of fish and their developmental stage.

## 2. Material and methods

### 2.1. Experimental details

We used groups of 10–60 Pacific blue-eyes (*Pseudomugil signifer*) with approximately three different body lengths (hereafter referred to as fish sizes): around 7.5 mm (small), around 13 mm (medium) and around 23 mm (large) (see the electronic supplementary material, figure S1). Because body size is related to the age of a fish [26], the three body lengths used in this study likely represent three distinct age classes. The largest fish (23 mm) constituted sexually mature individuals, although we observed no sexual behaviour in the trials. The fish were confined into a large shallow circular arena (760 mm diameter) and filmed from above at high spatial and temporal resolution. The positions of fish were subsequently tracked using DIDSON tracking software [27]. On average 86% of fish were identified and tracked in our experiments, which is a similar level of accuracy when compared with other studies that track large numbers of individuals [9].

### 2.2. Model

**Figure 1.**
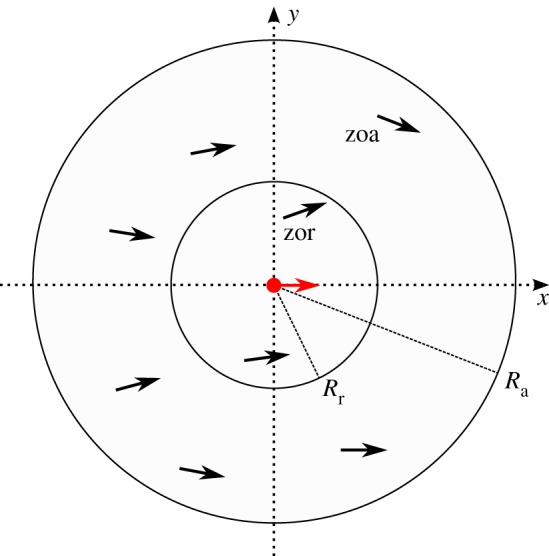
The illustration of the interaction parameters in the self-propelled particle model. The particle shown in red turns away from the nearest neighbours within the zone of repulsion (zor) to avoid collisions and aligns itself with the neighbours within the zone of alignment (zoa).

In our two-dimensional model, the fish are represented by *N* point particles at number density *ρ* and variable particle speed *v*_*i*_. The system undergoes discrete-time dynamics with a time step Δ*t*. The direction of motion of each particle (figure 1) is affected by repulsive or alignment interactions with other particles located inside the zone of repulsion (zor) or zone of alignment (zoa), respectively. Time evolution, therefore, consists of two steps: velocity updating and streaming (position update). In the first computational step, position of each particle (**r**_*i*_) is compared to the location of the nearest neighbours. The repulsion rule has an absolute priority in the model and is modelled as a typical collision avoidance [23,28] $\hat{\text{u}}{\left(t\right)}_{i}=-\sum_{j\neq i}^{n_{r}}{\text{r}}_{ij}(t)/|\sum_{j\neq i}^{n_{r}}{\text{r}}_{ij}(t)|$ with **r**_*ij*_=**r**_*j*_−**r**_*i*_ and *n*_r_ being a number of particles inside zor. The alignment rule similar to one used in the Vicsek model [29] takes into account velocities of all particles located inside the zone of alignment $\hat{\text{u}}{\left(t\right)}_{i}=\sum_{j=1}^{n_{a}}{\text{V}}_{j}(t)/|\sum_{j=1}^{n_{a}}{\text{V}}_{j}(t)|$ with *n*_a_ being a number of particles inside zoa. The velocities of particles are updated according to

S7 $${\text{V}}_{i}(t)=v_{i}(t)\hat{\text{u}}{\left(t\right)}_{i}R_{1}(\xi_{i}(t))R_{2}(\theta_{i}(t)),$$

with *v*_*i*_(*t*)=*v*_0_[*ψ*(*t*)]^*γ*^ defining the particle individual speed *v*_*i*_(*t*) based on the averaged local order *ψ*(*t*) inside both behavioural zones [30–33]. Here, *v*_*i*_(*t*) takes its maximal value *v*_*i*_(*t*)=*v*_0_ when velocities of particles inside zor and zoa are perfectly aligned *ψ*(*t*)=1 while the absence of local order *ψ*(*t*)=0 results in *v*_*i*_(*t*)=0. The exponent *γ* controls the sharpness of the speed change. Note that for any *γ* an isolated particle will move with maximal speed *v*_0_. The misaligning noise is introduced through a random rotation *R*_1_(*ξ*_*i*_(*t*)) of the resulting particle velocity according to a Gaussian distribution $P(\xi_{i}(t))=e^{-\xi_{i}^{2}(t)/2\eta^{2}}/\sqrt{2\xi_{i}(t)}\eta$, where *ξ*_*i*_(*t*) is a random variable and *η* is the noise strength.

Wall avoidance is modelled as a particle orientation adjustment through rotation *R*_2_(*θ*_*i*_(*t*)) of the particle velocity with a time-dependent turning rate *θ*_*i*_(*t*)=*v*_0_*ϕ*_*i*_(*t*)/*d*_*i*_(*t*). Here, *ϕ*_*i*_(*t*) is the angle between the heading of a fish and normal to a time-dependent point of impact on the wall [34,35]. Here, *d*_*i*_(*t*) denotes a distance from particle *i* to the impact point. Such construction of the rotating rate allows one to achieve strong damping at large distances from the wall and for smaller angles of approach of a collision point on the wall. At these conditions, its influence on particle motion is insignificant.

When the velocity update step is complete, the particle positions are updated by

2.1 $${\text{r}}_{i}(t+\Delta t)={\text{r}}_{i}(t)+{\text{V}}_{i}(t)\Delta t.$$

The model was parametrized based on the experimentally obtained data. The unit of length in our simulations is equivalent to the metric length used in the experiment. To set the unit of time, we choose a particle speed *v*_0_=*bv*^e^_0_, where *b* is the behavioural reaction time [36] of fish (*b*=0.05 s) and *v*^e^_0_ is the average speed in experiment. The integration time step was set to Δ*t*=1 for all simulations. The noise strength *η* was fixed at 0.1 for all trials. The exponent *γ* was set to 1 for all simulations. Fish of different size are modelled by scaling the size of the alignment zone with a factor *k* proportionally to experimentally measured differences in body lengths so that *k*=1, *k*=1.73 and *k*=3.07 correspond to small (7.5 mm), medium (13 mm) and large (23 mm) fish, respectively.

Total number of time steps in each run was 1×10^6^. The statistics was collected in the steady state and each characteristic of motion was calculated by averaging over five independent runs. The radius of the arena was fixed at *R*=380 for all simulations. The initial conditions for fish positions and velocities were chosen at random from the uniform distribution.

## 3. Results

**Figure 2.**
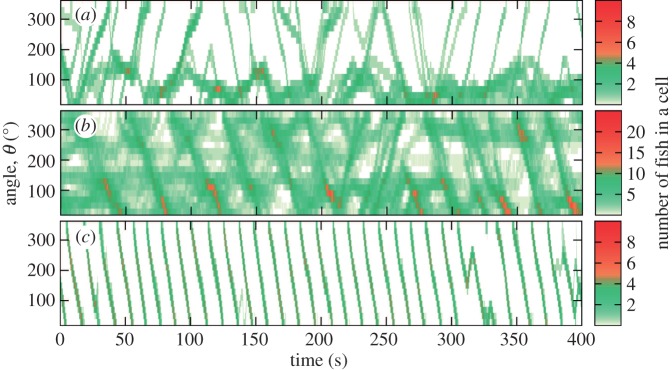
Example time evolution of the spatial distribution in arena at *N*=10 small fish (*a*), *N*=60 small fish (*b*) and *N*=10 large fish (*c*). Each cell in plots (*a*–*c*) denotes a 20° radial segment of the arena and 1 s of time. *θ* is the angle measured counterclockwise from the positive direction of the *x*-axis as defined by the camera position.

We first investigated the spatial distribution of fish in the arena. Small fish displayed limited collective motion. For example, 10 small fish tended to form a dispersed group, where most of the fish moved very little (figure 2*a*). Larger groups of 60 small fish showed slightly more collective motion, but not all fish moved in the same direction at the same time (figure 2*b*). By contrast, even small groups of large fish showed highly aligned collective motion (figure 2*c*).

**Figure 3.**
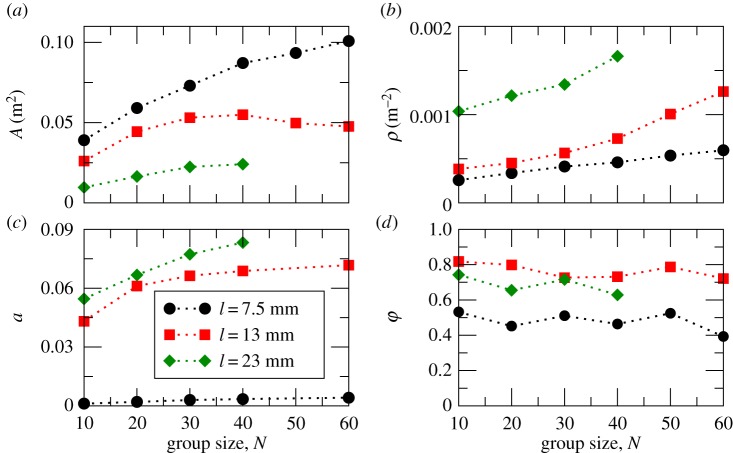
Statistical properties for groups of fish with three different body lengths: 7.5, 13 and 23 mm. (*a*) Area of a group *A*. (*b*) Number density *ρ*. (*c*) Packing fraction *a*. (*d*) Polar order parameter *φ*.

We calculated the average area, *A*, covered by the group using a convex hull algorithm (see the electronic supplementary material). The average value of *A* is plotted for different group and fish sizes (figure 3*a*). For all three fish sizes, larger groups occupied a larger area. The density, *ρ*=*N*/*A*, also increased with the number of fish in the group (figure 3*b*), suggesting that the fish pack closer together in larger groups. Figure 3*a* indicates that groups of small fish occupied a larger area than the groups of medium-size or big fish. This finding supports the results of the spatial analysis (figure 2); small fish were more dispersed over the arena. As a result, groups consisting of small fish were less dense (figure 3*b*) than groups of larger fish.

To better quantify the spatial arrangement of groups, we measured their packing fraction *a* (figure 3*c*). This is the ratio between the total body area of all fish in a group ($A_{f}=\sum_{i=1}^{N}A_{i}$) and the global area of a group (*A*): *a*=*A*_f_/*A*, where *A*_*i*_ is the body area of individual fish. For all body sizes of fish, packing fraction increased with group size. Groups of smaller fish had the lowest packing fraction ranging between 0.001 and 0.004 for groups of 10 and 60 individuals, respectively. By contrast, groups of medium-size and large fish had higher packing fractions of *a*>0.043 and *a*>0.054, respectively. The lowest packing fractions in groups of small fish are comparable to those observed in bird flocks [37,38], while the larger packing fractions approach those of some bacteria [39]. In physical systems, small values of *a* typically correspond to gases, while larger values (*a*>0.4) to liquids or crystals [40]. All packing fractions observed in our experiments, therefore, are comparable with an atomistic system in its gaseous state. At the same time, the large differences between packing fractions for small and medium-size fish and for small and large fish, reaching one order of magnitude (*t*-test, *p*<1×10^−6^), suggest possible differences in other statistical characteristics of the system for varying fish size.

We next characterized ordering in our system using the polar order parameter [29]

3.1 $$\varphi =\left\langle \frac{1}{N}\left|\sum_{i=1}^{N}\exp(ı\theta_{i})\right|\right\rangle ,$$

where ı is the imaginary unit, *θ*_*i*_ is the direction of motion of individual fish, and 〈⋅〉 denotes the time average. We should note that the polar order parameter is generally sensitive to the choice of confining geometry within which the agents move. For instance, for a small circular arena, the relationship between the radius of the arena and radial positions and speeds of the agents determines the maximum value of polarization that can be reached in the system. In our experiments, we used a large arena with a radius exceeding the average group width for large fish by more than three times. Large fish also occupied on average a radial segment of only 30° (figure 2*c*). Thus, we expect that the polar order parameter can provide meaningful results in characterization of alignment in our experimental system. Values of *φ* are plotted in figure 3*d* as a function of group and fish size. Small fish (7.5 mm) did not display much ordering for any group size with *φ*≈0.4–0.55, confirming previous observations (figure 2*a*,*b*). Groups of medium-size fish were the most ordered, with values of the order parameter ranging between 0.73 and 0.84 depending on group size. Large fish displayed slightly lower order than medium-size fish.

We then investigated the nature of the interactions between the fish using statistical mechanics. We started by looking at the pair distribution function (PDF) [28] which allowed us to study how the local density around each fish varied with respect to the average density in the system. It is defined by

**Figure 4.**
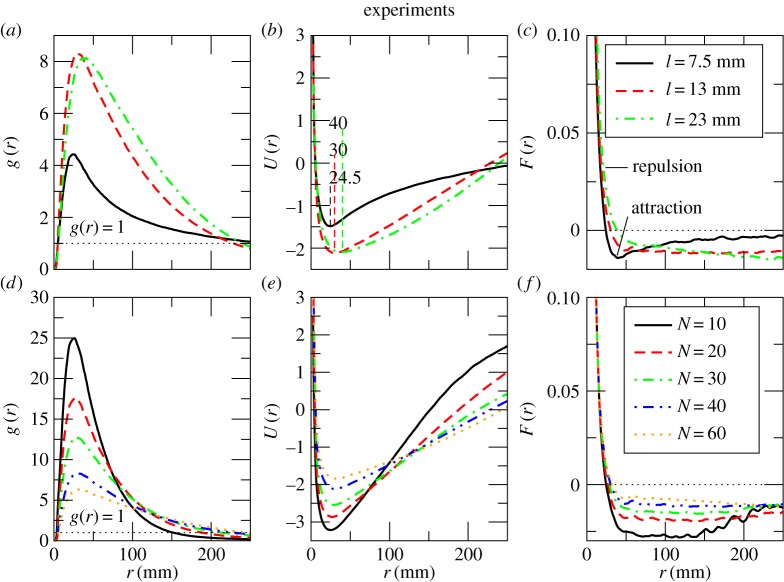
Statistical properties of fish in experiments. (*a*) Pair distribution function *g*(*r*) smoothed with 5-point moving average, (*b*) effective potential of the mean force of the interaction *U*(*r*) and (*c*) mean force of the interaction *F*(*r*) for small (7.5 mm) medium (13 mm) and large (23 mm) fish. (*d*) Pair distribution function *g*(*r*), (*e*) effective potential of the mean force of the interaction *U*(*r*) and (*f*) mean force of the interaction *F*(*r*) for medium-sized fish (13 mm) in groups of different size. The top row contains plots for different body size and the bottom row contains plots for different group size. The top and bottom legends correspond to plots in the top and bottom rows, respectively.

3.2 $$g(r)=\frac{1}{S(r)}\;\frac{1}{N(r)}\left\langle \sum_{i=1}^{N(r)}\sum_{j\neq i}^{N(r)}\delta (r-|{\text{r}}_{ij}|)\right\rangle ,$$

where *δ* is the Dirac delta function, |**r**_*ij*_| is the distance between fish *i* and *j*, *S*(*r*) is the surface area of a shell, *N*(*r*) is the number of fish inside a shell and 〈⋅〉 stands for the time average (see the electronic supplementary material for details). A set of PDF curves *g*(*r*) for various fish sizes is presented in figure 4*a*. Small fish tended to form aggregations with densities up to four times above the average density of the system and with a maximal half-radial width of more than 25 fish body lengths. For medium and large fish, the maximum density in a cluster was eight times larger than the average in the system. The size of the aggregation of medium and big fish was as large as 17 and 10 body lengths, respectively. Another notable difference between the three curves is the location of the local density peak. For small fish, the peak is at 24.5 mm, whereas for larger fish it is significantly shifted towards 30–40 mm. Figure 4*d* shows PDF plots for medium-sized fish at varying group size. For the smallest groups of 10 fish, the maximum density observed is 25 times above the system’s average density. The peak value of *g*(*r*) decreases with increasing group size (from 25 for groups of 10 fish down to 6.5 for the largest groups of 60 fish) while the position of the maximum remains unchanged at approximately 30 mm for all group sizes. The maximal half-radial width of the aggregation increases with group size from 145 mm for groups of 10 fish to 240 mm for groups of 60 fish.

The differences in the pair distribution function suggest that there is large variation in the underlying pair potential. In our system, in the absence of any external field, this potential is the effective potential of the mean force of the interactions between fish. Studies of active matter show that the resulting steady states of such systems often satisfy a Boltzmann distribution [5], even given that these active systems are essentially out of equilibrium. Here, we apply the opposite route: we start with the assumption that the steady-state configurations observed in the experiment are drawn from the Boltzmann distribution

3.3 $$P(r)=Z^{-1}\exp\left[-\beta U(r)\right],$$

where *β*=1/*k*_B_*T*=1 with a Boltzmann constant *k*_B_ and the system’s temperature denoted by *T*; Z=∫exp⁡[−βU(r)] dr is the partition function and *U*(*r*) is the effective potential of the interaction. In this work, the choice of the inverse temperature *β*=1 is arbitrary as this term is not related to thermodynamic temperature in our system. Instead, it accounts for temperature-like fluctuations in the system which we assume to take the same value for all three size classes of fish considered here. The fact that we observe large differences in the stationary distributions of different sized fish and different group sizes is related to the changes in motion of individuals when the configuration of neighbours changes. Nevertheless, the average number of individuals within each shell of the pair distribution function remains constant over the whole duration of an experiment, defining a steady state in our system.

To derive the effective potential of the mean force of the interactions, we use the direct Boltzmann inversion [22]

3.4 $$U(r)=-k_{B}T\ln g(r).$$

Figure 4*b*,*e* presents the effective potential energy profiles for different sized fish and different group sizes, respectively. Note that all the curves in both figures have practically the same slope for the decreasing part of the potential down to *U*(*r*)=−0.55. The minimum of these curves occurs at a greater distance for larger fish (figure 4*b*), indicating that larger fish have a larger repulsion zone. The increasing portions of the curves have similar slopes only for medium-sized and large fish (figure 4*b*). This suggests that fish of different body sizes and in groups of various size have similar repulsion strength (or collision avoidance potential) at short distances, but different attraction strengths towards neighbours. To get a conclusive picture of the variation of the interaction strength over the separation distance between the individuals, we calculate the mean force of the interaction *F*(*r*):

3.5 $$F(r)=-\frac{d}{dr}U(r).$$

In differently sized fish (figure 4*c*), the repulsive force (positive and short portion of the *F*(*r*) curves) is stronger than the attractive one (negative and long portion of the *F*(*r*) curves). In other words, repulsion is independent of body size and spans a much shorter distance than attraction. Constant attraction force at large distances in our system arises because fish can perceive conspecifics over the entire arena. In other systems, a change of the attraction force over distance could be used for identifying topological interactions between individuals. Previous studies [41] used specific channel confinement that mimics a geometrically frustrated anti-ferromagnet to distinguish between different types of interactions. Our approach is simpler and is not limited to specific experimental set-ups. For example, an abrupt change in the strength of attractive force would correspond to metric-type interactions.

**Figure 5.**
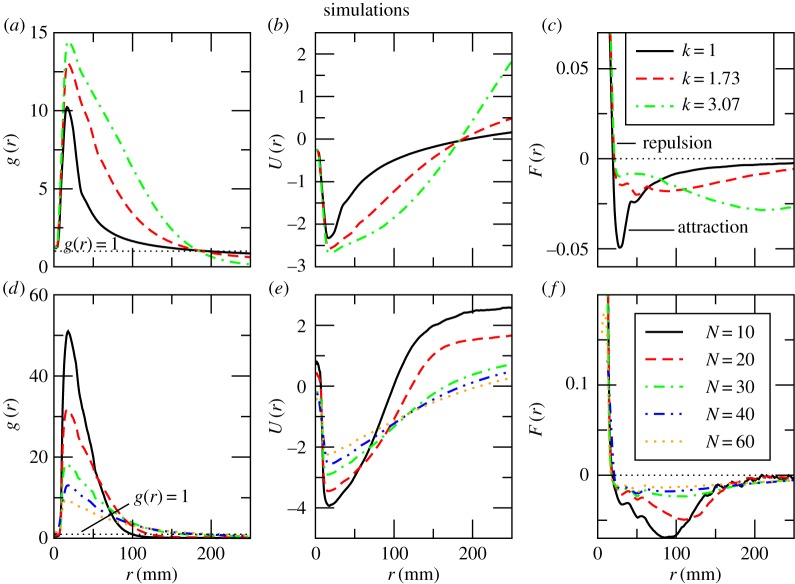
Statistical properties of a model system. (*a*) Pair distribution function *g*(*r*), (*b*) effective potential of the mean force of the interaction *U*(*r*) and (*c*) mean force of the interaction *F*(*r*) for small (*k*=1), medium (*k*=1.73) and large (*k*=3.07) simulated fish. (*d*) Pair distribution function *g*(*r*), (*e*) effective potential of the mean force of the interaction *U*(*r*) and (*f*) mean force of the interaction *F*(*r*) for medium-sized fish (13 mm) in groups of different size. The top row contains plots for different body size and the bottom row contains plots for different group size. The top and bottom legends correspond to plots in the top and bottom rows, respectively.

To validate the fish interactions established from the experiments, we simulated the collective motion of the fish using a two-dimensional metric self-propelled particle model that accounts for variable fish speed and geometrical confinement (see Methods for model description and the electronic supplementary material for details of other motion statistics). Figure 5*a*–*c* presents plots of PDF, effective interaction potential and mean force of the interaction for the three different sizes of simulated fish. Overall these qualitatively match the experimental data. Medium (*k*=1.73) and large (*k*=3.07) simulated fish form more dense groups than the small simulated fish (*k*=1) with a density of 12.5, 14 and 10 times above the average, respectively (figure 5*a*). The density peak is also observed at larger distances for bigger simulated fish. The repulsive force is practically the same for all three cases at any given distance (figure 5*c*) as the radii of the repulsion zone are constant for all fish sizes. The attractive portion of the *F*(*r*) curves has a complex shape as in the experiment indicating strongest attraction at *r*≈28, *r*≈90 and *r*≈200 mm for small, medium and large simulated fish, respectively. For all five group sizes of the medium sized simulated fish (*k*=1.73) the maximum of the local density is well above the system’s average (figure 5*d*) and is largest for groups of 10 fish (*g*(*r*)=52). All the curves cross the *g*(*r*)=1 line in the same order as in the experiment: the average half-radial width of groups of 10 and 60 simulated fish are around 100 mm and around 210 mm, respectively. As in the experiment, the repulsive force (figure 5*f*) decays with a distance and takes very similar values for all five cases. The attraction in groups of 10 and 20 individuals is maximal at *r*=90 and *r*=115, respectively, then decreases steeply with a distance and reaches zero at *r*≈190. For the other three curves (*N*=30, 40 and 60), the attractive force has a maximum at *r*≈100. At all intermediate distances, the dissipation is stronger in small groups of simulated fish which is in agreement with experiments.

## 4. Discussion

Although many recent studies have focused on understanding interactions between fish in schools [3,9,42], a detailed description of large systems consisting of tens or hundreds of individuals thus far remained a challenge. In analysis of such systems, the quality of fish tracking becomes a limiting factor, as many of the techniques traditionally used to study fish behaviour require high consistency of individual fish identities over time in order to reconstruct velocity or acceleration profiles of the individuals. When the long-time individual identities are not available, an approach that relies only on individuals’ positional data, such as Boltzmann inversion, can be useful for assessing interactions in large groups. The latter approach is also faster as it works with spatial data only, and the pair distribution function used in its first step allows also the extraction of useful information about aggregations and clustering in the system. Such approach can also potentially simplify fish tracking and post-processing of experimental data.

This approach, however, also has its limitations. The direct Boltzmann inversion has become a popular method to derive interaction potentials across different fields first of all due to its simplicity and general applicability. Even though this method has a straightforward nature, the potentials derived with the Boltzmann inversion are generally non-unique and can be state-dependent. They can also be influenced by long-range correlations or by the anisotropic nature of the interactions that is inherent to many living systems. Moreover, these potentials are effective as they may include multi-particle correlations (e.g. simultaneous response to positioning or movements of several conspecifics) and correlated contributions from the surroundings, such as geometrical confinement effects. Such potentials thus do not share the typical properties attached to equilibrium potentials and this limits their use mostly to qualitative description of the interactions. This also suggests that to draw a conclusive quantitative picture on how individuals interact in a group, a method like Boltzmann inversion needs to be accompanied by rigorous analyses of other motion statistics and/or by extensive computer simulations of the system of interest.

The challenges of using self-propelled particle models for modelling collective animal behaviour are also known [43,44]. The tractable models are usually oversimplified and too general and thus lack flexibility required to reflect the features of a particular phenomenon. In this work, we tried to overcome many of these limitations by parametrizing our simple self-propelled particle model with experimental data. Some of the simplifications, on the other hand, were introduced in the model deliberately, such as the absence of an explicit attraction rule for the interaction between the agents (see also Model section). We found the inclusion of the attraction rule unnecessary since a combination of fish–fish alignment and fish–wall interactions proved to be sufficient to reproduce the dynamics observed in experiments both statistically and visually [44]. We should note, however, that the effective cohesion detected in the model system by the direct Boltzmann inversion method is hence attributed to a combination of the inter-individual and agent–wall interactions. Even more detailed description of the system and possibly a better fit to experimental data could be achieved if mass and inertial forces of the individuals are taken into account. These features can be relatively easily implemented in a model which describes the motion of individuals by including friction and stochastic forces, e.g. active Brownian particle model with aligning interactions [45,46].

## 5. Conclusion

Using a combination of Boltzmann inversion and traditional statistical methods, we have inferred how fish (*Pseudomugil signifer*) interact in schools. Previous studies have applied a force-matching approach to infer the interactions of schooling fish from their movements [42]. Our method can infer these interactions directly from the static spatial distribution of individuals in groups. While repulsion forces had the same strength for different sized fish, attraction strength increased in larger fish, consistent with how a fish’s movement develops with age [25]. The interactions between fish also changed as a function of group size, as suggested by other studies [35]. Our model, refined on the basis of these observations, could capture the dynamics of schooling fish. These findings are also in line with the results of our previous study [44], where we used an observational test to cross-validate the model used in this study. We expect that our findings could also generalize to other species that exhibit schooling behaviour [47,48]. Application of the approaches used in statistical physics, coupled with informed models of collective motion, now allows us to shed more light on the intricacies of how individuals in groups interact.

## Supplementary Material

Supplementary Text

## Data accessibility

The datasets supporting this article are available from http://dx.doi.org/10.5061/dryad.8g0d0 [49].
