# Supplementary material for: Body size affects the strength of social interactions and spatial organization of a schooling fish (Pseudomugil signifer)
Source: R Soc Open Sci. 2017 Apr 26;4(4):161056. doi: 10.1098/rsos.161056 (PMC5414259; doi:10.1098/rsos.161056)
Supplement: Supplementary Text [file rsos161056supp1.pdf]

Supplemental Material for: Body size affects the strength of social interactions and spatial organisation of a schooling fish (*Pseudomugil signifer*)

Maksym Romenskyy,[1] James E. Herbert-Read,[1,2] Ashley J. W. Ward,[3] and David J. T. Sumpter[1]

[1]*Department of Mathematics, Uppsala University, Box 480, Uppsala 75106, Sweden*
[2]*Department of Zoology, Stockholm University, Stockholm 10691, Sweden*
[3]*School of Biological Sciences, University of Sydney, Sydney, New South Wales, Australia*

(Dated: February 21, 2017)

EXPERIMENTAL DETAILS

Materials

Pacific blue-eye fish (*Pseudomugil signifer*) were caught in hand nets from Narrabeen Lagoon, New South Wales, Australia (33°43′03 S, 151°16′17 E). Fish were kept in filtered freshwater in 150 l glass tanks at $22 - 25°$ and fed crushed flake food *ad libitum*. All fish were housed for at least three weeks prior to experimentation. The experimental arena was circular with a diameter of 760 mm. It was filled to a depth of 70 mm with aged and conditioned tap water. The arena was lit by fluorescent lamps and was visually isolated. For each trial, we randomly selected N fish ($N = 10, 20, 30, 40, 50$ or 60 for small and medium fish and $N = 10, 20, 30, 40$ for large fish) of similar size (see Fig. S1) from the housing tanks and placed them in the experimental arena. Fish were left to acclimate to the arena for at least five minutes, after which they were filmed for 15-20 minutes at 15 frames per second. We used a Logitech Pro 9000 camera placed orthogonally to the arena above its geometrical centre at a distance > 1 m minimising the radial distortion. The number of trials for each group size ranged between 3-10 (see Table I) due to limitations in the number of fish we could obtain for large or small body sizes. Because of the large numbers of fish we used for the experiment, we reused fish between trials. Fish were never used more than once per day and fish were used a maximum of 5 times.

Data collection and acquisition

Films were recorded in .mov format using original camera manufacturer software and subsequently converted to .avi using DirectShowSource and VirtualDub (v 1.9.2). The tracking was performed using DIDSON tracking program [S1]. The raw data consisted of x and y coordinates, fish identity and a time stamp. The accuracy of the tracking process was checked by projecting the raw tracking data onto experimental videos.

Figure S2 shows radial distribution of fish in the arena calculated as $g(R) = \rho(R)/\langle\rho\rangle$, where $\rho(R)$ is the density of fish in the circular shell of mean radius R and $\langle\rho\rangle$ is the average fish density in the system. Small fish $l = 7.5$ mm are

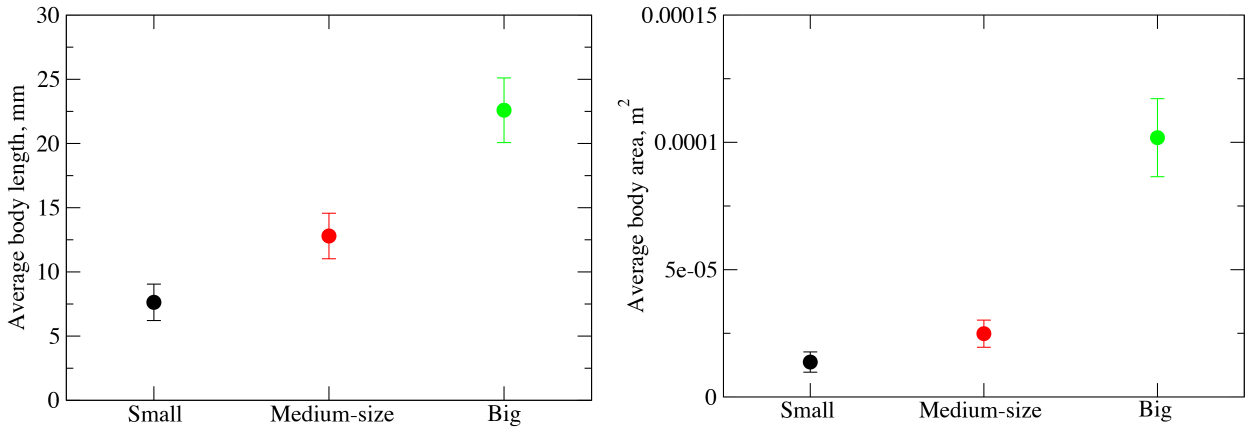

FIG. S1: Average fish body length ±1 s.d. for each size class (left). Average fish body area ±1 s.d. for each size class (right).

Group size	Average body length		
	7.5 mm	13 mm	23 mm
10	4	10	3
20	4	8	3
30	4	8	3
40	3	9	3
50	3	6	–
60	3	6	–

TABLE S I: Number of videos recorded for each group size and body size. Each film is 15-20 min long.

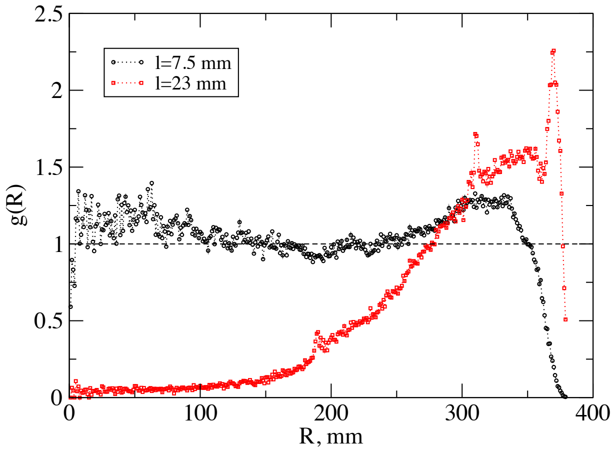

FIG. S2: Distribution of fish across the arena in radial direction ($N = 40$). 0 mm corresponds to the center of the arena; the wall is located at 380 mm from the center. The dotted black line at $g(R) = 1$ corresponds to completely homogeneous distribution of fish throughout the arena.

distributed more regularly throughout the tank as compared to large individuals $l = 23$ mm tending to move close to the arena wall.

MOTION STATISTICS ANALYSIS

Calculation of the shell area for the pair distribution function in confinement

When calculating the pair distribution function for confined systems particular care needs to be taken when the particles are located close to the wall, at a distance smaller than the radius of the largest shell. In experiments with fish this scenario is common (see Fig. S2 for illustration). In such situations only area of the shell lying inside the boundaries of the confining geometry should be considered. For all cases the shell area can be calculated as a difference between the areas of two neighbouring circles. The two latter areas for convenience can be computed separately as intersections excluding the areas outside the constraint. If confinement is circular (see Fig. S3), as is in our case, to find the intersection area we can use a formula for the circular segment of triangular height d' (excluding the height of the arced portion) and radius R' [S2]:

$$S(R', d') = {R'}^2 \cos^{-1}(d'/R') - d'\sqrt{R'^2 - d'^2}, \tag{S1}$$

In the simplified case that we consider here (with only one circle for the pair distribution function) we have two such segments, one for the circular confinement (experimental arena) with a radius R and hight d_1, and another one for the shell boundary of the pair distribution function, having radius r and height d_2. The heights are calculated as

$$d_1 = \frac{d^2 - r^2 + R^2}{2d}, \tag{S2}$$

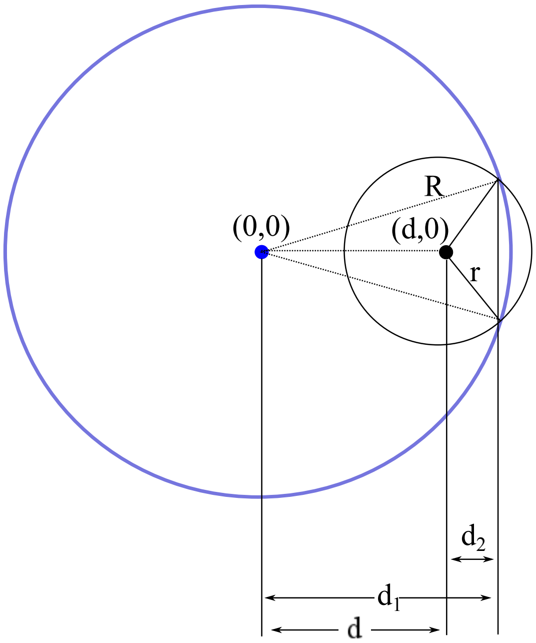

FIG. S3: Illustration of the intersection of the pair distribution function circle r with confining circle R. Only one circle for the pair distribution function is drawn for simplicity.

and

$$d_2 = \frac{d^2 + r^2 - R^2}{2d}. \tag{S3}$$

To calculate the total area of the intersection Eq. (S1) needs to be solved two times, once for each segment. Thus, combining Eqs. (S1-S3) we get

$$S(r') = S(R, d_1) + S(r, d_2) = \\ = r^2 \cos^{-1}\left(\frac{d^2 + r^2 - R^2}{2dr}\right) + R^2 \cos^{-1}\left(\frac{d^2 + R^2 - r^2}{2dR}\right) - \frac{1}{2}\sqrt{(-d + r + R)(d + r - R)(d - r + R)(d + r + R)}. \tag{S4}$$

Figure S4 shows a plot of the pair distribution of particles for highly homogeneous system in circular confinement (distribution of particles is uniform). The black curve displays a clear linear decay of $g(r)$ with increasing inter-particle separation distance r. This represents the case where the area outside the constraint has also been included in calculations. The red curve represents the case when all the shell areas have been calculated with the method described above. This shows highly regular distribution $g(r)$ of particles for all separation distances r ($1:1$ relation between the local shell density and average density in the system).

Calculation of the surface area of a fish group and the body area of an individual fish

The surface area of a group was calculated for every frame of a video. First, we computed a convex hull C of a set of N points representing geometrical centres of fish bodies [S3]. The convex hull is defined by

$$C \equiv \sum_{i=1}^{N} \lambda_i p_i : \lambda \geq 0 \text{ for all } i \text{ and } \sum_{i=1}^{N} \lambda_i = 1, \tag{S5}$$

where i is the point (fish) index of a point p_i and λ is the non-negative weight coefficient. The resulting convex hull gives the identities of the vertices of a polygon as an output. The area A of this polygon can be computed as [S4]

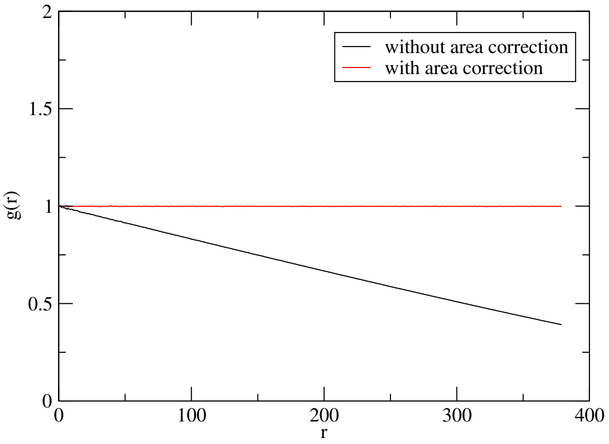

FIG. S4: Pair distribution function $g(r)$ for a homogeneous test system constituting of 1000 particles (average over 5×10^6 positional configurations), $R = 380$.

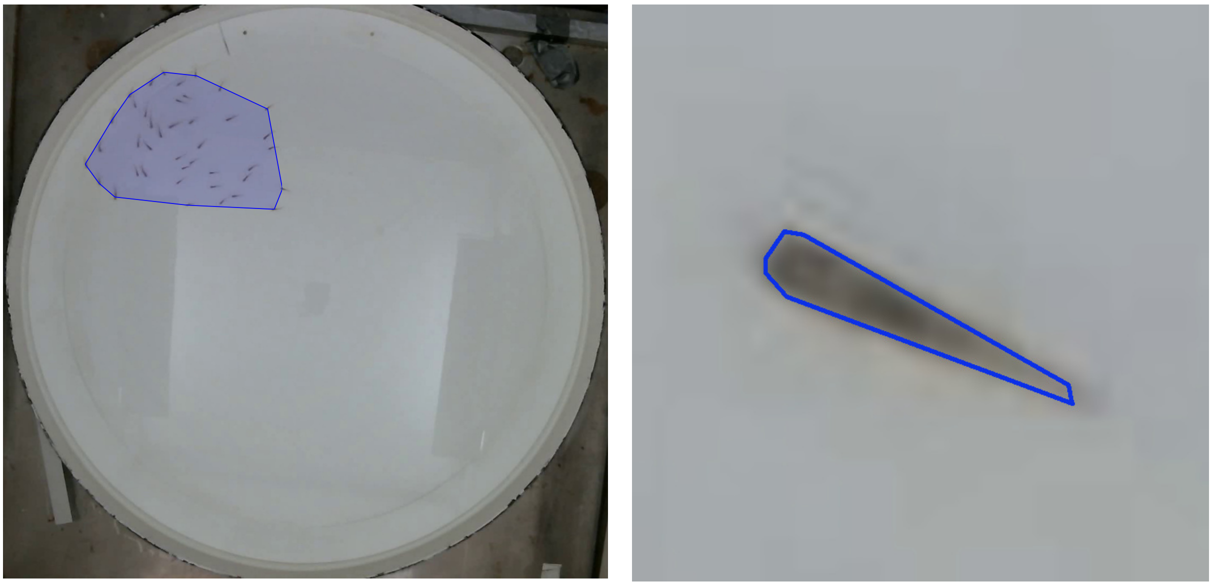

FIG. S5: Complex shapes obtained from the experimental data. Left: Typical polygon returned by a convex hull algorithm. Right: Fish body shape estimated as a convex shape. The displayed frame is taken from a video for 40 medium-size fish. The frame on the left has been scaled for clarity purposes.

$$A = \frac{1}{2}\sum_{i=1}^{N}(x_i y_{i+1} - x_{i+1} y_i), \tag{S6}$$

where x_i and y_i represent coordinates of the vertices of a polygon. The last vertex (x_{N+1}, y_{N+1}) is assumed to be the same as the first one, so $(x_{N+1}, y_{N+1}) = (x_1, y_1)$ and the polygon is closed.

The body area of an individual fish was computed for every identified individual in every frame of a movie. All edge pixels forming a shape of an object (fish) were identified based on the preset weighted intensity threshold. The resulting shape was a polygon with N vertices corresponding to a number of edge pixels with intensity values above the threshold. The area of this complex shape was computed with the same method as for the surface area of a group.

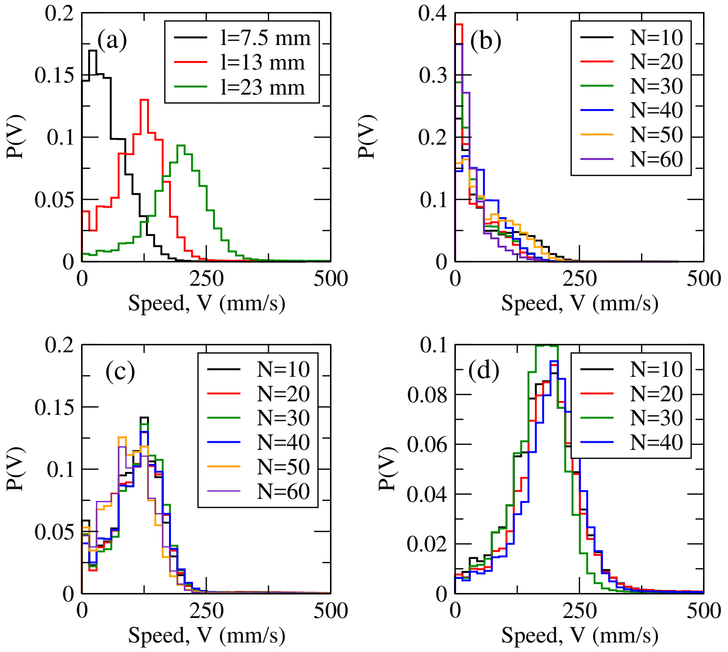

FIG. S6: Experimental speed distributions for: (a) three sizes of fish in groups of 40 individuals, (b) small (l=7.5 mm), (c) medium (l=13 mm) and (d) large (l=23 mm) fish at variable group size.

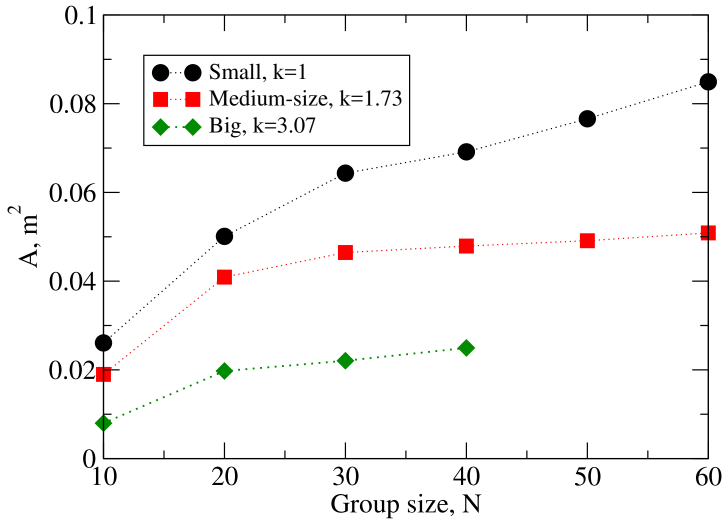

FIG. S7: Average area of a simulated group of fish for three size classes: small ($k = 1$), medium-size ($k = 1.73$), and big (3.07) fish. Simulation parameters as stated in the main text, Methods section.

Additional motion statistics

Figure S6(a) shows speed distributions for fish of three average body lengths at fixed group size (N=40). Small fish (l=7.5 mm) most of the time have speeds below 100 mm/s and very often within a range of 0-20 mm/s. Very rarely small fish have speed above 200 mm/s. For medium-size individuals the distribution is much wider and has a peak at $V = 125$ mm/s. It spans up to $V \approx 250$ mm/s and has another minimum at 0-20 mm/s. For large fish the number of events when the individuals are stationary or barely move decreases further and the peak is observed at $V \approx 200$. Figures S6(b)-(d) show speed distributions for small, medium and large fish and various group sizes. All histograms for the same body size practically overlap. Therefore, while speed regime of fish depends strongly on body size it is not effected by the number of individuals in a group.

Figure S7 shows the average group area occupied by simulated fish as a function of number of individuals in a

group. For all three sizes of simulated fish, the group area increases with group size, in agreement with experimental results (Fig. 3(a), main text). For all group sizes, big fish form densest groups. Conversely, groups of small fish occupy much larger area as compared to the other two size classes (medium-size and big fish).

[S1] N. O. Handegard and K. Williams, ICES J. Marine Sci.: Journal du Conseil **65**, 636 (2008), URL `http://icesjms.oxfordjournals.org/content/65/4/636.abstract`.

[S2] E. W. Weisstein, *Circle-circle intersection*, MathWorld – A Wolfram Web Resource. `http://mathworld.wolfram.com/Circle-CircleIntersection.html`.

[S3] C. B. Barber, D. P. Dobkin, and H. Huhdanpaa, ACM Trans. Math. Softw. **22**, 469 (1996), ISSN 0098-3500, URL `http://doi.acm.org/10.1145/235815.235821`.

[S4] P. Bourke, *Calculating the area and centroid of a polygon*, `http://paulbourke.net/geometry/polygonmesh/`.
